# Polyradiculitis and encephalomyelitis in the same patient following a SARS-CoV-2 vaccination

**DOI:** 10.1186/s42466-022-00177-w

**Published:** 2022-03-28

**Authors:** Josef Finsterer

**Affiliations:** Neurology and Neurophysiology Center, Postfach 20, 1180 Vienna, Austria

## Letter to the Editor

We read with interest the article by Stefanou et al. about a 47yo male who developed Guillain–Barre syndrome (GBS) followed by acute, demyelinating encephalomyelitis (ADEM) after a vaccination with the Ad26.COV2.S vaccine (Johnson & Johnson) [1]. For GBS, the patient received intravenous immunoglobulins (IVIG) and for ADEM steroids [1]. The patient made a partial recovery until discharge to a rehabilitation unit [1]. It was concluded that “it would be worth investigating the implication of autoantibodies against ACE-2 and neuropilin-1 in future cases presenting with neurological symptoms following vaccination against SARS-CoV-2” [1]. The study is appealing but raises concerns that need to be discussed.

We do not agree with the statement that “neurological adverse events following immunisation against SARS-CoV-2 have been shown to be rare” [1]. There are in fact studies showing that the frequency of side effects to SARS-CoV-2 vaccinations is low but there are also indications from real world data that neurological adverse reactions to anti-SARS-CoV-2 vaccines are common [2, 3]. There is also increasing proof that not only mild or moderate side effects but in fact severe or even fatal adverse reactions can occur [4]. One reason for the delayed recognition of severe side effects is that often a causal relation between the vaccination and the timely neurological compromise is not suspected. A second reason could be that all available vaccines were approved without extensive exploration of their safety profile, why severe side effects might have been missed.

We also do not agree with the statement that “neurological adverse reactions following immunisation against SARS-CoV-2 have been shown less frequent in patients undergoing vaccination against SARS-CoV-2 as compared to patients with COVID-19” [1]. First, neurological involvement in SARS-CoV-2 infections is known for a longer period than adverse reactions to anti-SARS-CoV-2 vaccinations, which are available now (by the end of January 2022) for just over one year. This might be one reason why there is the impression that complications following a SARS-CoV-2 infection are more prevalent than complications following an anti-SARS-CoV-2 vaccination. Second, it is more convenient to report and discuss complications of a disease than complications of a vaccine. Reporting adverse reactions following a vaccination requires standing up against the producer and those who approved the compound.

Furthermore, we are not convinced that the index patient had ADEM. Since the patient had received IVIG prior to taking the images provided in figure 1 and since IVIG can be complicated by hyperintensities within neuronal structures [5, 6], it is conceivable that the lesions shown in the cerebellar peduncle, at the C4/5 and T1 levels, and along the thoracic spine are in fact reactions to IVIG. IVIG has been previously shown to cause osmotic demyelination syndrome [7]. This particular reaction against IVIGs could be more extensive in vaccinated patients than among those without the vaccination. Therefore, we should be informed about the electrolyte levels and the renal function parameters during hospitalisation to rule out or confirm extra-pontine myelinolysis.

We also should be told why the left peduncular lesion did not manifest clinically and it should be explained why the patient had a T6 sensory level and not a C5 level since there was an enhancing lesion at C4/5.

Overall, the interesting report has some limitations that call into question some of the results and their interpretation. Mild to severe adverse reaction to any of the available SARS-CoV-2 vaccines are more frequent than usually propagated. Anti-SARS-CoV-2 vaccinations are not safe for everyone, and IVIG may be responsible for further deterioration of a GBS developing 4 weeks after the vaccination.

## Data Availability

All data are available from the corresponding author.
